# Lithium induced hypercalcemia: an expert opinion and management algorithm

**DOI:** 10.1186/s40345-022-00283-3

**Published:** 2022-12-22

**Authors:** Zoltan Kovacs, Peter Vestergaard, Rasmus W. Licht, Sune P. V. Straszek, Anne Sofie Hansen, Allan H. Young, Anne Duffy, Bruno Müller-Oerlinghausen, Florian Seemueller, Gabriele Sani, Janusz Rubakowski, Josef Priller, Lars Vedel Kessing, Leonardo Tondo, Martin Alda, Mirko Manchia, Paul Grof, Phillip Ritter, Tomas Hajek, Ute Lewitzka, Veerle Bergink, Michael Bauer, René Ernst Nielsen

**Affiliations:** 1 Psychiatry, Research and Treatment Program for Bipolar Disorder, Aalborg University Hospital, Mølleparkvej 10, 9000 Aalborg, Denmark; 2 Department of Clinical Medicine, Aalborg University, Aalborg, Denmark; 3 Department of Endocrinology, Aalborg University Hospital, Aalborg, Denmark; 4 Steno Diabetes Center North Jutland, Aalborg, Denmark; 5 Department of Psychological Medicine, Institute of Psychiatry, Psychology and Neuroscience, King’s College London & South London and Maudsley NHS Foundation Trust, Bethlem Royal Hospital, Monks Orchard Road, Beckenham, Kent, BR3 3BX UK; 6 Department of Psychiatry, Queen’s University, Kingston, ON Canada; 7 Medical Faculty Brandenburg Theodor Fontane, Neuruppin, Germany; 8 Department of Psychiatry, Psychotherapy, Psychosomatics and Neuropsychiatry, Kbo-Lech-Mangfall-Klinik Garmisch-Partenkirchen, Auenstr.6, 82467 Garmisch-Partenkirchen, Germany; 9 Department of Neuroscience, Section of Psychiatry, Università Cattolica del Sacro Cuore, Rome, Italy; 10 Department of Psychiatry, Fondazione Policlinico Universitario Agostino Gemelli IRCCS, Rome, Italy; 11 Department of Adult Psychiatry, Poznan University of Medical Sciences, Poznan, Poland; 12 School of Medicine, Department of Psychiatry and Psychotherapy, Technical University of Munich, 81675 Munich, Germany; 13 Charité-Universitätsmedizin Berlin and DZNE, 10117 Berlin, Germany; 14 University of Edinburgh and UK DRI, Edinburgh, EH16 4SB UK; 15 Institute of Psychiatry, Psychology and Neuroscience, King’s College, London, UK; 16 Copenhagen Affective Disorder Research Center (CADIC), Psychiatric Center Copenhagen, Copenhagen, Denmark; 17 Department of Medicine, University of Copenhagen, Copenhagen, Denmark; 18 Mood Disorder Centro Lucio Bini, Cagliari, Italy; 19 Rome McLean Hospital, Harvard Medical School, Rome, Italy; 20 Department of Psychiatry, Dalhousie University, Halifax, Canada; 21 National Institute of Mental Health, Klecany, Czech Republic; 22 Section of Psychiatry, Department of Medical Sciences and Public Health, University of Cagliari, Cagliari, Italy; 23 Department of Pharmacology, Dalhousie University, Halifax, NS Canada; 24 Unit of Clinical Psychiatry, University Hospital Agency of Cagliari, Cagliari, Italy; 25 Mood Disorders Center, Ottawa, ON Canada; 26 University of Toronto, Toronto, ON Canada; 27 Department of Psychiatry and Psychotherapy, University Hospital Carl Gustav Carus, TU Dresden, Dresden, Germany; 28 Department of Psychiatry, Icahn School of Medicine at Mount Sinai, New York City, NY USA; 29 Department of Psychiatry, Erasmus Medical Center, Rotterdam, The Netherlands

**Keywords:** Lithium, Side-effects, Bipolar disorder, Affective disorder

## Abstract

**Background:**

Lithium is the gold standard prophylactic treatment for bipolar disorder. Most clinical practice guidelines recommend regular calcium assessments as part of monitoring lithium treatment, but easy-to-implement specific management strategies in the event of abnormal calcium levels are lacking.

**Methods:**

Based on a narrative review of the effects of lithium on calcium and parathyroid hormone (PTH) homeostasis and its clinical implications, experts developed a step-by-step algorithm to guide the initial management of emergent hypercalcemia during lithium treatment.

**Results:**

In the event of albumin-corrected plasma calcium levels above the upper limit, PTH and calcium levels should be measured after two weeks. Measurement of PTH and calcium levels should preferably be repeated after one month in case of normal or high PTH level, and after one week in case of low PTH level, independently of calcium levels. Calcium levels above 2.8 mmol/l may require a more acute approach. If PTH and calcium levels are normalized, repeated measurements are suggested after six months. In case of persistent PTH and calcium abnormalities, referral to an endocrinologist is suggested since further examination may be needed.

**Conclusions:**

Standardized consensus driven management may diminish the potential risk of clinicians avoiding the use of lithium because of uncertainties about managing side-effects and consequently hindering some patients from receiving an optimal treatment.

## Background

Lithium is the gold standard in recurrence prevention in patients diagnosed with bipolar disorders (Licht 2012). Data has also supported a substantial reduction of suicides and suicide-related mortality (Lewitzka et al. 2015; Malhi et al. 2017; Tondo et al. 2001), although newer randomized trials have not been able to replicate this effect when compared to placebo (Katz et al. 2022), possibly related to low mean lithium concentration, patient sample and a short follow-up period (Manchia et al. 2022). Furthermore, lithium is indicated in the treatment of patients with unipolar depressive disorder as an augmenting strategy in combination with antidepressants or as a monotherapy—especially with a highly recurrent illness course (Tiihonen et al. 2017). Lithium as a maintenance treatment for bipolar disorder has demonstrated to be superior to placebo and at least comparable to relevant active comparators, even when the comparator was tested under enriched study conditions, i.e., the randomized patients were responders to acute treatment of the drug (Licht 2012). Despite the high level of evidence supporting the effectiveness of lithium in the treatment of unipolar and bipolar disorders and its continued position as a first line treatment in various practice guidelines, its use has diminished over the last decades (Malhi et al. 2021). This underutilization seems particularly at odds with the strength of the evidence.

Due to the low therapeutic index of lithium, therapeutic drug monitoring is required along with laboratory monitoring of thyroid and renal functions and by measurements of calcium levels. The laboratory monitoring should be performed in combination with regular clinical assessments of potential side-effects, such as gastrointestinal symptoms, tremor, polyuria and cognitive dulling (McKnight et al. 2012). The symptoms related to high calcium in the blood may depend on the level of hypercalcemia and how rapid the increase has been. Slightly increased levels of calcium or slowly rising levels may be associated with mild or no symptoms. The clinical symptoms associated with high calcium include CNS: forgetfulness or mild to severe cognitive dysfunction; gastrointestinal tract: abdominal pain, constipation, nausea and vomiting; urogenital tract: frequent urination, increased thirst, kidney stones; musculoskeletal system: joint and muscle pain, and fatigue. Due to symptoms being mostly vague, and could be caused by other conditions, including mental disorders, the use of standardized measurements, including plasma calcium levels, are essential when treating patients. Further, elevated plasma PTH levels have shown to be associated with higher risk for cardiovascular mortality, presence and severity of depression in elderly (Hagström et al. 2009; Hoogendijk et al. 2008).

To facilitate the safe use of lithium and to prevent clinicians from avoiding using lithium when clinically indicated due to uncertainties about how to practically manage side-effects, clinical guidelines for treatment management are needed (Tondo et al. 2019).

In the present paper, we provide an overview of the homeostasis of the calcium levels relevant to lithium treatment and propose a step-by-step algorithm to guide clinicians on the initial management of emergent hypercalcemia in patients during lithium treatment.

### Prevalence of Calcium and PTH disturbances in lithium-treated patients

A systematic review and meta-analysis found that average levels of calcium and PTH in patients treated with lithium are increased by 10% compared to controls (McKnight et al. 2012). Patients exposed to lithium have an increased risk of developing hypercalcemia (OR 13.45; 95% CI 3.09, 58.55; p < 0.001) compared to non-exposed (Meehan et al. 2018). Albert et al. (2013) found a relatively high proportion of lithium-treated patients with elevated PTH (8,6%) and calcium levels (24,1%) (Albert et al. 2013), while Meehan et al. (2015) reported a higher prevalence of hyperparathyroidism (18%) in patients exposed to lithium (Meehan et al. 2015). For comparison, the prevalence of primary hyperparathyroidism in the general population has been between 0.78% and 1.07% across Latin-America, United States and Europe (Khan et al. 2017). Further, the incidence of primary hyperparathyroidism in the general population has been increasing in the recent decades (Abood & Vestergaard 2013), and differences exist between developed and developing countries regarding prevalence, incidence, and clinical presentation (Yadav et al. 2020)*.* Primary hyperparathyroidism is also predominantly seen in women with a ratio of as much as 3:1 over men (Vestergaard & Mosekilde 2003). Especially in women after menopause, the difference becomes more apparent, and the increase seen in primary hyperparathyroidism in recent decades have mainly been seen among women (Abood & Vestergaard 2013). Whether this is by chance or stems from physiological differences such as the fact that the decline in estrogen levels following menopause may increase the calcium loss from the skeleton putting strain on the parathyroid glands to increase PTH to counter this remains elusive.

At the moment little is known on the effects of sex regarding calcium disturbances and lithium, However, from available evidence, it seems that the majority of patients with lithium induced hyperparathyroidism are women (Meehan et al. 2020). However, it remains unclear if this is the result of the fact that more women than men may receive treatment with lithium for say depression or is a consequence of the mechanisms mentioned above.

### Overview of calcium and PTH homeostasis

**Fig. 1:**
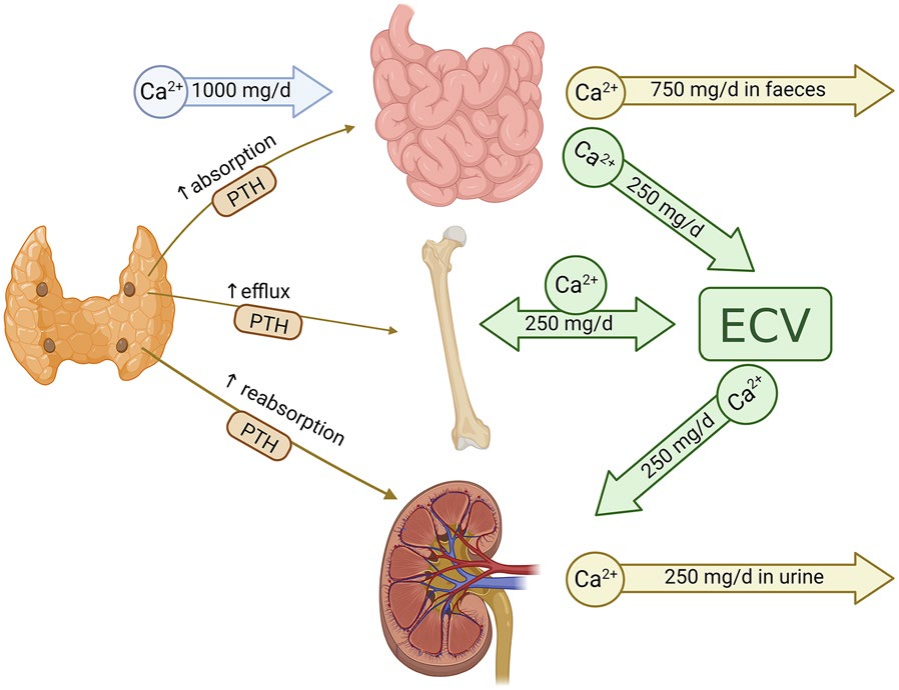
The basics of calcium homeostasis. *Mg/d* milligram per day, *PTH* Parathyroid hormone, *ECV* extracellular volume

Figure 1 shows the basics of the calcium homeostasis. Most of the calcium in the body is concentrated in the skeleton (> 99%), while small amounts are present in the blood (Vestergaard 2015).

Parathyroid hormone (PTH) is secreted from the four parathyroid glands located behind the thyroid gland in the lower neck and increases plasma calcium directly by increasing the efflux from the skeleton and the reabsorption of calcium from the urine (thus decreasing the loss), as well as indirectly through increasing the activation of vitamin D. Vitamin D is ingested with food or formed in the skin during exposure to sunlight. Vitamin D increases the absorption of calcium in the intestine and decreases its loss in the urine. Calcium is also absorbed passively in the intestine from ingestion.

PTH secretion is regulated by the calcium sensing receptor (CaSR), which responds to an increase in plasma calcium levels by decreasing PTH, whereas a decrease in plasma calcium levels triggers an increase in PTH levels under normal circumstances.

Thus, the system is rather complex and changes in one component may thus not always lead to changes in other parts of the system due to compensatory mechanisms and counter-regulations as described in more detail below.

Hypercalcemia may be divided into: Hypercalcemia with increased PTH (i.e. PTH above normal range) and hypercalcemia with decreased PTH (i.e. PTH below normal range).

Hypercalcemia with increased PTH stems from primary or tertiary hyperparathyroidism. Lithium increases PTH most often associated with hyperplasia of all four parathyroid glands. Lithium associated hyperparathyroidism may differ in several ways from primary hyperparathyroidism (Berger et al. 2013; Mak et al. 1998; Meehan et al. 2020). Some of these differences may also account for the potential bone-protective properties of lithium (Köhler-Forsberg et al. 2022).

There are a number of causes for hypercalcemia with decreased PTH including malignancies, over intoxication with calcium and vitamin D or vitamin V to sarcoidosis, etc. For a comprehensive list please see Etiology of hypercalcemia—UpToDate (Shane et al. n.d., https://www.uptodate.com/contents/etiology-of-hypercalcemia).

### Interaction between lithium and calcium homeostasis

Ionized lithium (Li^+^) resembles ionized calcium (Ca + +) and acts as a calcilytic by antagonizing the CaSR (Nemeth 2002). In this way, the CaSR senses the plasma calcium level as potentially being too low, and PTH secretion is increased, subsequently leading to an increased efflux of calcium from the skeleton, an increased reabsorption in the kidneys and an increased calcium absorption in the intestine. However, this sequence may be counterbalanced by other parts of the system such as calcium intake, vitamin D level, PTH receptor sensitivity, vitamin D receptor sensitivity, physical activity, and kidney function. If the PTH levels increase as a result of chronic lithium exposure, usually, all four parathyroid glands develop hyperplasia over a period of years (Vestergaard et al. 2000). However, the increase in size and activity may be unevenly distributed with one or more glands dominating. This increase in size and function may lead to autonomous secretion of PTH no longer responsive to plasma calcium changes.

The main cause of increased calcium in lithium-treated patients is therefore typically associated with hyperplasia of all four parathyroids. This contrast with primary hyperparathyroidism (Walker & Silverberg 2018), which is most often (90% or more) caused by an adenoma of only one of the four parathyroid glands (Vestergaard et al. 2000) and has consequences for the management of primary hyperparathyroidism in lithium-treated patients. In patients with an adenoma, if surgical treatment is indicated, usually only the affected parathyroid gland needs to be removed and the remaining can easily restore normal calcium metabolism. However, in patients with hyperplasia affecting all four parathyroid glands, surgically removing either all four or three glands plus parts of the fourth may result in significant symptomatic hypoparathyroidism (Bilezikian et al. 2014).

Little is known on predictors of disturbances in calcium metabolism with lithium over time. Some patients develop early disturbances and in some these may regress, whereas others may not develop disturbances in calcium metabolism despite lengthy lithium treatment. Due to the complex nature of the calcium metabolism, differences in the various components and receptors and vitamin D status may all either help to maintain the equilibrium as seen in most patients or may not be able to maintain the equilibrium.

### Clinical practice guidelines on monitoring calcium and PTH in lithium-treated patients

Several authors (Gitlin 2016; McKnight et al. 2012; Shapiro & Davis 2015) and clinical practice guidelines recommend calcium monitoring during lithium treatment, more specifically assessment at baseline and at 6 and 12 months and then yearly, or more frequently if clinical symptoms such as polyuria, polydipsia, constipation, or fatigue are reported (Malhi et al. 2017). Data on monitoring practices among health care professionals from 24 countries generally showed compliance with practice guidelines, revealing that 80% of respondents assess plasma calcium levels before the start of lithium. However, during the maintenance phase, 16% of health professionals did not monitor plasma calcium levels, with 68% having assessed plasma calcium levels 1–3 times, and 16% having assessed the levels ≥ 4 times (Nederlof et al. 2018)*.*

Broome and Solorzano recommended determining lithium levels in case of confirmed concurrent elevations of plasma levels of PTH and calcium to rule out acute lithium intoxication as an etiological factor. In the absence of clinical symptoms and with only mildly elevated calcium levels, they recommended monitoring calcium levels at an interval of 6 to 12 months and continuing lithium treatment (Broome & Solorzano 2011).

Lehmann and Lee (2013) recommended monitoring calcium levels more frequently and observing for clinical symptoms in cases of mild asymptomatic hypercalcemia and absence of PTH level elevation while continuing lithium therapy. Furthermore, in severe cases with elevated plasma levels of both PTH and calcium, they recommended consultation with and eventually referral to an endocrinologist (Lehmann & Lee 2013).

A decision regarding continuation of lithium treatment, as for other interventions, should always be weighted between side-effects, patient and clinician’s opinion and severity of the disorder, effect of the treatment and previous treatments (Luby & Singareddy 2003). Although evidence is lacking, lithium dose reduction may be an easy initial strategy in order to attempt preventing further progression of the alterations of calcium and PTH levels, unless clinically contraindicated. In cases where changes in calcium or PTH levels are observed, discontinuation or continuation of lithium should be discussed between patient and the psychiatrist with the possible opinion of an endocrinologist describing possible long-term effects of increased calcium and PTH.

### Hypercalcemia management algorithm

**Fig. 2:**
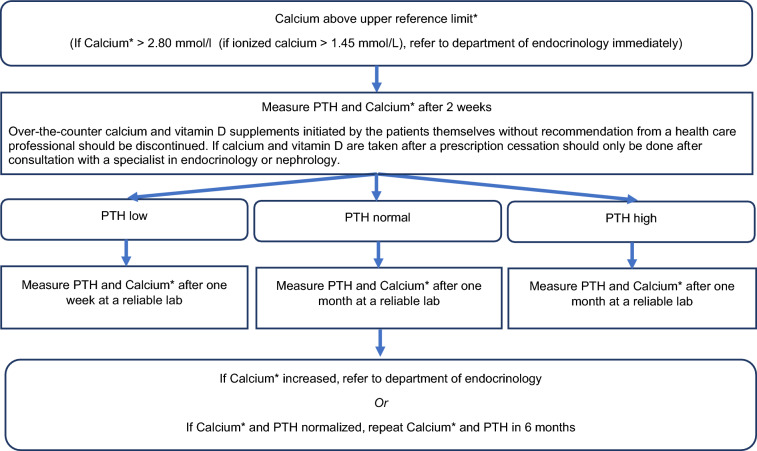
Hypercalcemia management algorithm (*albumin-corrected plasma calcium levels)

A standardized plan across clinical practices for the early management of abnormalities of calcium and PTH plasma levels is lacking. This situation led to the following algorithm being developed in collaboration with specialists trained in psychiatry and endocrinology (Fig. 2).

### Rationales behind the decision tree

The rationale behind the decision tree is to provide an easy and hopefully intuitive path to reach a comprehensive diagnosis and management of lithium-treated patients with evidence of abnormal calcium metabolism.

Step one is the assessment of hypercalcemia measuring calcium in plasma as either level of total plasma calcium level, plasma albumin adjusted calcium level or ionized plasma calcium level (the latter measured as the value at the actual pH-value or at a standardized pH of 7.40). If the plasma calcium level is very high, this may be dangerous for the patient and in accordance with recommendations from the American Society for Bone and Mineral Research (Bilezikian et al. 2014) we suggest a cut-off for immediate consultation with the department of endocrinology or a calcium metabolic specialist at 2.80 mmol/l for total and albumin adjusted calcium levels and at 1.45 mmol/l for ionized calcium levels.

The following is a short overview of the most common possible causes of hypercalcemia to provide the setting for the decision tree. Increased plasma calcium levels may be the result of high ingestion of calcium as supplements which need to be paused in case of hypercalcemia. Vitamin D supplements, either as native cholecalciferol or ergocalciferol or activated vitamin D such as calcitriol or calcifediol may also explain the high calcium level and should likewise be paused; however, pausing activated vitamin D such as calcifediol and calcitriol should only be performed after consulting an endocrinologist. Over-the-counter cholecalciferol and ergocalciferol rarely cause hypercalcemia. Treatment with thiazides should also be discussed with a nephrologist since these may decrease calcium excretion in the urine and lead to increase in calcium levels. As it may take some time for a new equilibrium to establish, a new measurement should usually be performed after two weeks.

To ensure correct handling of blood samples and measuring of plasma calcium, measurement at a hospital or other reliable laboratory is recommended as compared to blood samples at the general practitioner, if possible.

If PTH and calcium are both high, this is indicative of primary or tertiary hyperparathyroidism. This is often a stable condition, i.e., plasma calcium stays at the same level and does not increase presenting no immediate risk to the patient. To establish the diagnosis at least two measurements of PTH and plasma calcium are needed. If PTH and calcium are increased at both measurements a primary hyperparathyroidism is likely and an endocrinologist should be consulted. Further examinations initiated by the endocrinologist may include measurements of bone density with a bone scan (DXA) to check if calcium loss from the skeleton has resulted in osteoporosis and to establish if parathyroid surgery may be indicated. Of note, however, a recent Danish registry-based retrospective cohort study showed that while the risk of osteoporosis was higher in patients with bipolar disorder, this risk was decreased in patients treated with lithium compared to those not receiving lithium (Köhler-Forsberg et al. 2022). Further, renal imaging may be indicated to assess for nephrocalcinosis or kidney stones, which may also present with an indication for parathyroid surgery. Parathyroid surgery may be performed as minimal invasive surgery if one gland is affected, which is determined by ultrasound and supplemental sestamibi scintigraphy or other imaging modality. If more than one gland is affected, surgery may be more extensive.

If plasma calcium normalizes, but PTH is increased, this suggests secondary hyperparathyroidism. This may be the result of vitamin D deficiency, reduced kidney function (as indicated by increased serum creatinine) or several other conditions such as secondary hyperparathyroidism resulting from treatment with lithium or treatment with antiresorptive drugs for osteoporosis (bisphosphonates, denosumab etc.), from use of estrogen or estrogen/progestin replacement therapy in women, obesity, or low ingestion of calcium in the diet. A detailed description covering this topic is beyond the scope of this paper. Routine screening for abnormalities in PTH levels are not indicated if plasma calcium is normal or in cases where vitamin D is low or kidney function is reduced. This is due to the large individual variations in PTH levels and sensitivity on the vitamin D receptor, calcium sensing receptor and PTH receptor and the low diagnostic yield.

If PTH is normal in case of abnormal plasma calcium, this may reflect measurement problems such as sampling errors when blood is collected or acid–base disturbances. In the presence of alkalosis in e.g., patients with chronic obstructive pulmonary disease (COPD), plasma calcium may be increased as follows from the Henderson-Hasselbalch equation, and vice versa in acidosis (e.g., reduced kidney function may result in low plasma calcium). In these cases, the condition is not acute and new measurements can be made after one month. Repeated measures are justified by random fluctuation in early primary hyperparathyroidism where plasma calcium may normalize temporarily but is captured on reassessment. Thus, our recommendation is re-evaluation after six months.

A low PTH may be indicative of underlying malignancy or vitamin D intoxication, high ingestion of calcium supplements or sarcoidosis. Malignancies may lead to hypercalcemia through efflux of calcium from the skeleton either through bone metastases (often lung or breast cancer) or through the production of substances that may mimic PTH (PTHrP), often seen in hematological malignancies such as multiple myeloma or lymphomas. Moreover, production of activated vitamin D may be associated with lymphomas and sarcoidosis (Bilezikian et al. 2018; Donovan et al. 2015). In all these cases, swift action may be required, and re-measurements need to be assessed within a week.

Some combinations of plasma calcium and PTH levels have not been covered in the above mentioned as they rarely occur and likely need consultation with an endocrinologist.

## Conclusions

Abnormalities in plasma calcium and PTH levels homeostasis are frequently seen in patients treated with lithium, but a standardized and practical approach guiding clinicians on how to monitor and manage the abnormalities and their causes in the psychiatric setting are hitherto lacking. Here we present an evidence-based consensus driven management algorithm intended to provide a helpful tool for clinicians monitoring long-term lithium treatment. Besides assuring the detection and proper handling of relevant calcium abnormalities, the algorithm will likely diminish the potential risk of premature discontinuation of lithium treatment in patients who benefit from lithium, due to treatment emergent hypercalcemia. Finally, the algorithm may reduce the risk of clinicians avoiding the use of lithium in patients who would otherwise benefit due to uncertainties about management of this potential often treatable side effect.

## Data Availability

All data are published.
